# Natural History, a Master Class

**DOI:** 10.1371/journal.pbio.1001496

**Published:** 2013-02-26

**Authors:** Andy Dobson

**Affiliations:** Eno Hall, Princeton University, Princeton, New Jersey, United States of America

## Abstract

Andrew Dobson on the inestimable value, and beauty, of the artful field journal.

[Fig pbio-1001496-g001]Many people think we're entering a golden age of genomics, with technological breakthroughs yielding an explosion of data along with unprecedented insights into the genes and molecules that underlie life. Personally, I find it all a bit dull and uninspiring. Perhaps this cynicism stems from a conversation I once had with a fanatical, eye-popping cladist who proudly told me, “We don't need to save those tropical forests, we've already got samples of most of their DNA in the museums!” This perspective is slowly giving rise to the significantly deranged belief that we don't need to worry about the loss of biodiversity: we'll simply recreate it from stored DNA. The current cult of genomics and its ominous trickle-down effects on high-school biology teaching means it's sometimes easy to lose sight of the biology that surrounds us. Even so, there's something about the natural world and its creatures that still sparks a deep fascination and likely inspired many of us to study biology in the first place. *Field Notes on Science and Nature* reminds us why we find nature so appealing and just how much fun getting into the field can be.

**Figure pbio-1001496-g001:**
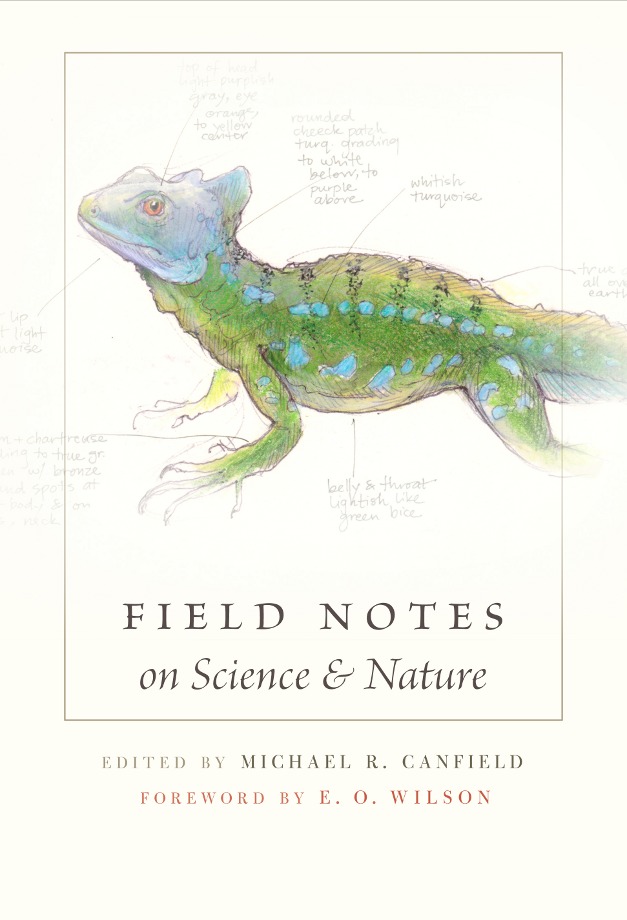
Canfield MR, editor (2011) Field Notes on Science and Nature. Cambridge, MA: Harvard University Press. 296p. ISBN 978-0674057579 (hardcover). US$27.95.

In many ways this book allows us to paraphrase Robert Hughes's definition of great art: “Great field notes are simultaneously of their time and timeless!” It's a wonderful book and one that can be both read instructively and also dipped into for pure pleasure. It contains insights into both the scientific process and method, but also provides lessons on how to look at the world from different perspectives—lessons that it is never too late (or early) to learn. These lessons come from the real masters: George Schaller, renowned conservationist and field biologist, tells us how notes from all-day follows of lions aggregated to become the raw data of scientific publications and the background material for his classic early books, *The Serengeti Lion* and *Golden Shadows, Flying Hooves*; Bernd Heinrich, the ultimate nature writer and physiologist, describes how several of his books stemmed from unusual observations noted in his field journals—observations which suggested something more profound had to be explained—whence *Winter World* and *The Mind of the Raven*, two of the finest works of natural history ever.

I have to confess to being an unresolved bibliophile. In the multiple weeks and months that I've been slowly relishing and reviewing this book, I've had the pleasure of using the web to locate a wonderful, first-edition copy of Frank Chapman's *My Tropical Air Castle*, G. Evelyn Hutchison's *The Ecological Theater and the Evolutionary Play*, and several older books describing early visits to the Serengeti and other parks in Tanzania and Kenya—sheer bibliophile bliss! Tragically, it has also been a self-indulgent excuse for failing to write the other reviews, articles, and grant proposals I should have been writing. But books like these turn on its head J.B.S. Haldane's battle cry: “My job is not to read the literature, my job is to write it!” There are times when the right book makes it well worth sitting back and taking time to absorb the skills and insights that can only be conveyed through someone else's writing.

The best nature writing is a wonderful art form. I can think of no example of it being successfully converted into film. This book provides many powerful glimpses into the techniques used to assemble deep understanding of the workings of nature. It cogently illustrates that while a central set of guidelines are followed by nearly everyone, each field worker develops their own idiosyncrasies that ultimately help them to achieve different insights, and these are often distilled into the finest of nature writing and illustrating. This contrasts with the work of “professional science writers,” who are often, to my taste, overstylized and too removed from the experience, the real sense of being there you get from reading “real scientists” like Heinrich, Terborgh, Wilson, Chapman, or Darwin. All too often the postmodern scaffolding of “Modern Writing 101” dominates the prose of many professional science writers, their work assembled like the over-busy travel agenda it took to organize all those interviews, the imagery as heavy as the meals consumed while the interviews took place, and the uncritical reverence for the interviewee as obsequious and hollow as the thanks delivered when the scientist picked up the tab for lunch. I personally blame the Pulitzer prize committee for this, as they seem unable to differentiate insight from long-windedness. The new ground rules seem to be “More is less, unless you want a Pulitzer!” (Hmm, I've just set myself up for some really bitchy reviews of anything I write in the future! Pah! C'est la vie!) Although, it is important to remember that there are many science writers whose work I really admire—David Quammen instantly springs to mind. They also perform the vital task of communicating complex science to the broader public, which is an area in which most scientists fail spectacularly.

*Field Notes on Science and Nature* includes a very insightful chapter by Piotr Naskrecki on “Note-Taking for Pencilophobes,” which provides a useful introduction to the multiple ways now available for collecting data on handheld devices in the field. My one concern here is that I will find it hard to generate the sense of wonder and memory when I go back and look at old electronic records. I would advise people to collect electronic data in conjunction with their field notebooks. I do not wish to appear a Luddite here; I can see the power of new techniques as much as any graduate student or postdoc (my iPod is plugged in as I'm typing this on my Android tablet), but one of my deepest worries is that a generation of scientists is being trained to believe they can understand everything they need to know about ecology and evolution without ever leaving the lab bench or computer cluster. This will create a generation that will at best never produce any eye-watering prose, or worse a generation that may let whatever biodiversity was around when they were born trickle away during the course of their lifetime. Ironically, we're living in a year when a novel about working in a tax-assessment office is hailed as a major literary work with deep insights into the human condition, so the bar is set fairly low. Erick Greene makes this point in his fine chapter on keeping a field journal. When he set this as an exercise for his upper-level ecology class, students complained that they'd signed up for ecology not “creative writing”! Yet the examples he includes of field notes from the class eloquently illustrate that the students soon found that keeping a field journal taught them to look at the world in a very different way; they were before long as far from creative writing as they could get, amazed at how much the world around them changed if they simply sat back and took the time to observe it in a consistent fashion. Greene and several of the other authors provide important tips on what to include in your field journal. I particularly liked the idea of pasting-in an envelope to keep track of receipts for field expenses (mine usually dissolve when my field clothes enter the wash!). Jenny Keller and Jonathan Kingdon provide fantastic introductions on how to sketch biology, and how to find a balance between figures and text that will allow you to recapture huge details of what you have seen in later analyses.

My own field notes point to a couple of things that are missing from the book. In the days when we tried to capture images of nature on Kodachrome 64 and Fuji Velvia, I would keep long lists of film canister numbers and shots between 1 and 36 that kept account of what I'd been photographing, the location, and the exposure. These helped me develop a future understanding of which exposures worked or, more often, failed in different light conditions. Nowadays our digital cameras record all this information for us, and we can get instant gratification and confirmation of the correct setting simply by examining the viewing screen. But this means I am less likely to make lists in my notebooks of the location of each shot, so there will be a diminished future joy of looking back through the notebooks and remembering the details of why I took particular sets of pictures. Curiously, several authors in the book argue against the use of photographs and prefer taking notes and sketches. I would again argue for a mixture. My own field notebooks also contain pages of equations, sketches of graphs, poems, songs, and drafts of speeches for politicians. None of the authors here admit to any of these vices, although several have their own eccentricities that make the many illustrated plates a delight to observe. Indeed the quality of the plates is so fine, it suggests to me that a larger electronic archive should be made of field books of great naturalists. This could match the large collection of film and photographs kept in Arkive (www.arkive.org): film and photographs shot by the great wildlife photographers but never used in film or publications, usually due to space constraints. In many cases, this material is our last and only chance to observe certain species in the wild. The complete field notes of the naturalists who write in this book need to be archived for similar reasons; they may well be the last records we have of the field behavior of many species.

This book is a *vade mecum* for the aspiring writer, providing hints and insight into different approaches that a wonderful collection of nature writers and illustrators have used in their work. These will be of inestimable value to many cohorts of professional science writers (if only it had appeared sooner!), while providing wonderful insights into why humans are so inspired by nature and find multiple deep, creative, and moving ways to express this delight and interest. All field biologists should own a copy of the book and dip into it regularly. All heads of departments and high school headmasters should buy multiple copies (I encouraged my chair to buy 30 copies). It is a timeless book that can always be given with pleasure as a prize to your best pupils. It will only be received with delight and will continue to provide insight and guidance as long as there are scientists who take pleasure in going into the field to study the natural world. This is nature, resplendent in detail and rich in field lore.

**About the Author**

Andy Dobson is serving a life sentence for ecology at Princeton University. His parole conditions allow him to teach students about parasitology in Panama and the ecology of savannas in East Africa. In alternate years he takes a new cohort of graduate students on a total-emersion tropical ecology course in the Neotropics. A plea bargain with the NSF and the NIH permits him to undertake research on pathogens in the Serengeti, in salt marshes along the coast of California, and in the eyes of house finches in the backyards of New England. More recently he has become interested in how food webs will respond to climate change, particularly in the High Arctic.

